# Magic-Formula-Based Feedforward Velocity Control of a Magnetically Coupled Wall-Climbing Robot

**DOI:** 10.3390/s26217063

**Published:** 2026-11-08

**Authors:** Minsu Kim, Hoyeon Cho, Sungkeun Yoo, Taegyun Kim

**Affiliations:** 1 Department of Mechanical Engineering, BK21 FOUR ERICA-ACE Center, Hanyang University, 55 Hanyangdaehak-ro, Ansan 15588, Gyeonggi, Republic of Korea; minsoo0828@hanyang.ac.kr (M.K.); zxcss456@hanyang.ac.kr (H.C.); 2 College of Engineering, Hongik University, 94 Wau-San-ro, Mapo-gu, Seoul 04066, Republic of Korea

**Keywords:** wall-climbing robot, magnetic adhesion, magic formula, feedforward compensation, path tracking

## Abstract

This study presents a model-based feedforward method for reducing velocity errors during the vertical travel of a magnetically coupled wall-climbing robot. Permanent magnets on opposite sides of the wall maintain magnetic coupling, while a spring-preloaded linkage adjusts the drive-wheel normal force. A dedicated test bench was used to measure the traction–slip characteristics of the urethane roller–acrylic contact at normal forces of 20 and 30 N. Magic Formula models were fitted to the measurements. Representative slip magnitudes were calculated from the models and the ascent force balance using a nominal equivalent resistance of the robot. These fixed magnitudes were applied to both ascent and descent through direction-dependent wheel-velocity commands. In experiments without longitudinal velocity feedback, the compensation reduced the absolute errors in mean velocity and travel distance, averaged over all tested conditions, by approximately 76% and 69%, respectively. The method was also integrated with proportional–integral velocity feedback and pure pursuit path tracking and assessed in repeated ascent–descent trials. The results demonstrate the error-reduction capability of the fixed feedforward compensation under the tested conditions.

## 1. Introduction

Wall-climbing robots can perform inspection, maintenance, cleaning, and nondestructive testing on vertical and inclined surfaces of large structures, including bridges, storage tanks, ships, and building facades [1,2,3]. These robots are useful for repetitive tasks in locations that are difficult or hazardous for workers to access. During vertical travel, gravity opposes ascent and assists descent, while the normal force available at the wheels is constrained by the adhesion mechanism and the allowable surface load. For surfaces such as acrylic panels and glass, increasing the normal force to obtain sufficient traction may cause damage. The surface material, contamination, roughness, and local geometry also affect the wheel–wall contact. Under these conditions, longitudinal slip may cause the wheel peripheral speed to exceed the robot’s travel speed during ascent, whereas during descent, the robot’s travel speed may exceed the wheel peripheral speed. The resulting mismatch introduces velocity and travel-distance errors, induces drift in wheel odometry, and degrades path tracking. Quantifying the traction–slip relationship under the actual wheel–surface contact conditions is therefore essential for accurate vertical motion.

The adhesion mechanism is a key factor for the safety and mobility of wall-climbing robots. For ferromagnetic structures, magnetic wheels and crawlers incorporating permanent magnets have been widely used to maintain adhesion [4,5,6,7,8]. In these designs, magnetic attraction directly affects the normal force at the wheel or track contact. Changes in magnet arrangement, magnet–wall gap, wall thickness, and surface curvature can therefore affect adhesion, rolling resistance, and available traction. Consequently, the overall adhesion force and the normal force at the drive wheels often cannot be treated as independent design variables.

The robot considered in this study uses magnets mounted on the bodies of the drive and passive modules located on opposite sides of the wall. Magnetic attraction couples the modules across the wall, and a spring-preloaded compliant linkage adjusts the normal force at the drive wheels. This arrangement distinguishes the roles of magnetic adhesion and wheel loading and allows the wheel normal force to be adjusted under fixed magnetic coupling conditions. Therefore, it provides a platform for evaluating the effect of the normal force on wheel–wall traction and incorporating the measured contact characteristics into wheel velocity commands.

Kinematic models and path-tracking controllers for wheeled mobile robots (WMRs) commonly assume pure rolling without slip. When friction is insufficient or the required traction is high, the commanded wheel motion can differ from the actual robot motion. Dynamic models and tracking controllers for wall-climbing robots have incorporated magnetic forces, gravity, friction, and drive torque [9,10]. However, including these forces does not necessarily account for the nonlinear relationship between longitudinal traction and slip. For example, the obstacle-crossing model developed by Fan et al. includes magnetic, gravitational, and frictional forces but assumes no lateral slip and pure rolling in its kinematic formulation [10].

Approaches to slip in WMRs include the explicit modeling of contact dynamics and the estimation or compensation of slip as an unknown disturbance. Tian and Sarkar incorporated longitudinal and lateral traction forces into a dynamic model and developed regulation and turning controllers under slip conditions [11]. Chen combined a disturbance observer with prescribed-performance control to address skidding, slipping, and input disturbances [12]. Wu and Wang incorporated estimated slip disturbances into a virtual reference trajectory rather than directly adding compensation terms to the control input [13]. Fuzzy slip compensation [14] and tracking control combining a drive-unit model with slip compensation [15] have also been reported. These studies address motion control in the presence of slip, whereas the present study uses an experimentally identified wheel–wall contact model to generate velocity commands for vertical ascent and descent.

Pacejka’s Magic Formula provides an empirical representation of nonlinear traction–slip characteristics [16]. It can capture the initial slope at low slip, peak traction, and saturation or force reduction at larger slip using a small set of parameters. These parameters can be estimated from force and motion measurements obtained on test rigs or during vehicle operation [17,18]. Load-dependent changes in the effective rolling radius and slip have also been reported for a small mobile platform [19], highlighting the importance of characterizing contact under relevant normal loads. For the present robot, the required relationship describes the traction–slip behavior of the robot’s urethane roller–acrylic contact.

Traction models have also been incorporated into slip control for climbing and crawler robots. Uth et al. used the Magic Formula to describe wheel–surface traction in an offshore crawler and investigated slip control with a disturbance observer along with mechanical modifications [20]. Their use of a contact model for slip regulation is closely related to the present work. Their slip controller addresses excessive slip, whereas this study uses an experimentally identified steady-state traction–slip model to compensate for velocity errors during vertical travel.

This study develops and evaluates feedforward velocity compensation based on measured wheel–wall contact characteristics. A dedicated test bench reproduces the robot’s urethane roller–acrylic contact, and Magic Formula models are identified from traction–slip measurements at two normal forces. The models and the ascent force balance are used to select a representative slip magnitude for each normal force. The same magnitude is applied to ascent and descent using different wheel-velocity conversion equations. The compensation values are fixed before the travel experiments and are not adjusted using the travel results. Their effect is evaluated by comparing travel with and without compensation in the absence of longitudinal velocity feedback. The combined use of feedforward compensation, proportional–integral (PI) velocity feedback, and pure pursuit path tracking was evaluated in repeated ascent–descent experiments.

The remainder of this paper is organized as follows. Section 2 describes the robot structure and wheel normal-force adjustment mechanism. Section 3 presents the traction–slip test bench, model identification results, and the calculation of representative slip ratios. Section 4 describes the feedforward velocity compensation and integrated control structure. Section 5 presents the vertical travel and path-tracking experiments and discusses their limitations. Section 6 concludes the paper.

## 2. Design of the Magnetically Coupled Wall-Climbing Robot

### 2.1. System Configuration and Magnetic Coupling

**Figure 1:**
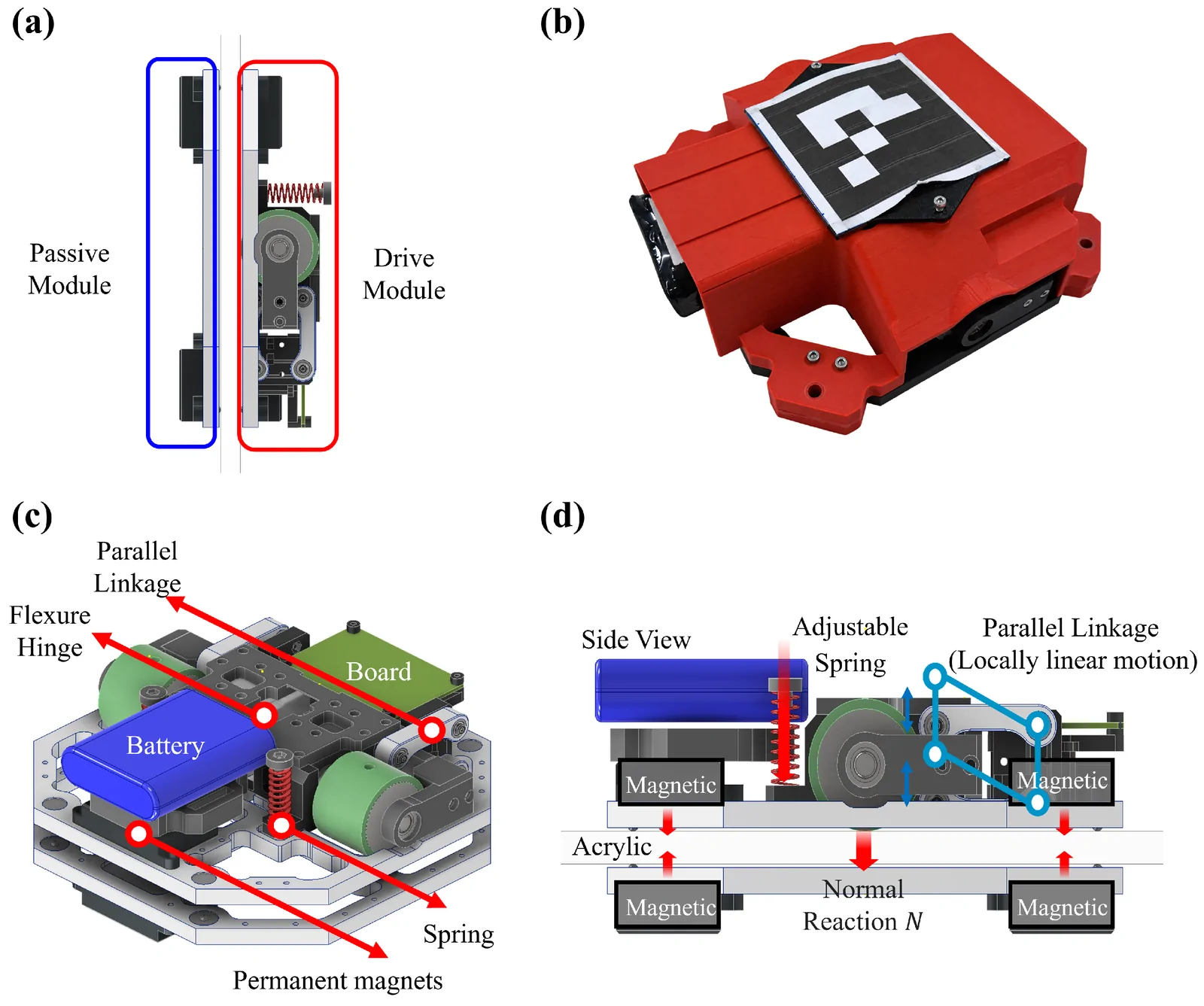
Configuration of the magnetically coupled wall-climbing robot. (**a**) Drive and passive modules magnetically coupled across the wall; (**b**) photograph of the robot prototype; (**c**) diagram of the robot components; (**d**) side view of the robot structure.

The wall-climbing robot consists of a drive module and passive module positioned on opposite sides of the wall (Figure 1a). The drive module has two independently driven wheels, while the passive module follows its motion on the opposite side through magnetic coupling. The robot is therefore designed for use on panel structures accessible from both sides, such as windows, acrylic walls, and thin partitions. The drive and passive modules have masses of 1.50 kg and 0.55 kg, respectively, yielding a total moving mass of 2.05 kg. Each module has two permanent magnets mounted on its body. The magnetic attraction can be adjusted by varying the gap between the opposing magnet surfaces. Four ball plungers on the drive module and six on the passive module maintain the clearance between the module bodies and the wall and restrict excessive tilting toward the wall under magnetic attraction.

Body-mounted magnets maintain wall adhesion, while spring preload adjusts the normal force at the drive wheels. This permits wheel-load adjustment under fixed magnetic coupling conditions, although the magnetic coupling force and wheel normal forces remain mechanically coupled. The primary design parameters are listed in Table 1.

### 2.2. Wheel Normal-Force Mechanism and System Integration

Each drive wheel is actuated using a servo actuator (XC430-W150-T, DYNAMIXEL, ROBOTIS, Seoul, Republic of Korea). The wheel contact element is a urethane roller (ROHAKN40-10-30, MISUMI, Tokyo, Japan) with a diameter of 40 mm and a Shore A hardness of 50. Rollers with the same specifications were used in the traction–slip test bench described in Section 3 to provide comparable roller contact characteristics in the test bench and the robot.

Each wheel module consists of a parallelogram four-bar linkage, an adjustable compression spring, and a flexure hinge (Figure 1c,d). The linkage connects the robot body to the drive wheel and permits a limited displacement in the wall-normal direction while approximately maintaining the orientation of the wheel module. Adjusting the compression of the spring located in front of the drive motor changes the wheel normal force. The spring force and wall reaction can generate a moment that tilts the wheel in the camber direction. The flexure hinge is designed to mitigate the effect of this moment on wheel alignment. The left and right wheel modules are structurally separate, allowing the normal force at each wheel to be adjusted individually.

The relationship between spring compression and wheel normal force depends on the flexure elasticity, magnetic attraction, and load distribution between the wheels and ball plungers. Therefore, this relationship was experimentally calibrated using a separate load-calibration fixture. The spring compression was varied incrementally to determine the settings corresponding to the target normal forces. These settings were used to adjust the spring preload prior to each test. The target normal forces were 20 N and 30 N per wheel. Load-cell measurements showed deviations from the target values within ±0.3 N. At the fixed 16mm gap between opposing magnet faces, the nominal magnetic attraction $F_{m}=82.8\;N$ acts on each module with equal magnitude and opposite direction. Under quasi-static wall-normal equilibrium,

$$F_{z,L}+F_{z,R}+R_{D}=F_{m},\;R_{P}=F_{m},$$

where $F_{z,L}$ and $F_{z,R}$ are the left and right wheel normal forces. The quantities $R_{D}$ and $R_{P}$ are the sums of the normal reactions at the four drive-side and six passive-side ball plungers, respectively. For equal wheel normal forces of 20N and 30N, $R_{D}$ is approximately 42.8N and 22.8N, respectively, while $R_{P}$ remains approximately 82.8N. These totals do not imply equal loading of individual ball plungers. At the nominal fixed magnetic gap, increasing spring preload transfers part of the drive-side contact load from the ball plungers to the wheels. The calculated total normal reaction at the drive-side ball plungers is positive at both wheel-load settings, consistent with shared support by the wheels and ball plungers in the nominal configuration.

Wheel velocity commands are generated by an external PC and transmitted over Wi-Fi to an ESP32-based controller, which relays them to the left and right drive motors. The robot pose is measured using an external vision system with ArUco markers [21]. Reference markers positioned at the four corners of the wall define the wall coordinate frame. The pose of the marker attached to the robot, expressed relative to this frame, is used to calculate the robot pose vector

(1) $$q_{a}={\left[\begin{array}{ccc} x_{a} & y_{a} & \theta_{a} \end{array}\right]}^{T},$$

where $x_{a}$ and $y_{a}$ are the lateral and vertical positions in the wall coordinate frame, respectively, and $\theta_{a}$ is the robot heading angle in the wall plane. Details of the camera arrangement and experimental setup are provided in Section 5.1.

## 3. Traction–Slip Characterization and Model Identification

### 3.1. Test Bench and Experimental Procedure

**Figure 2:**
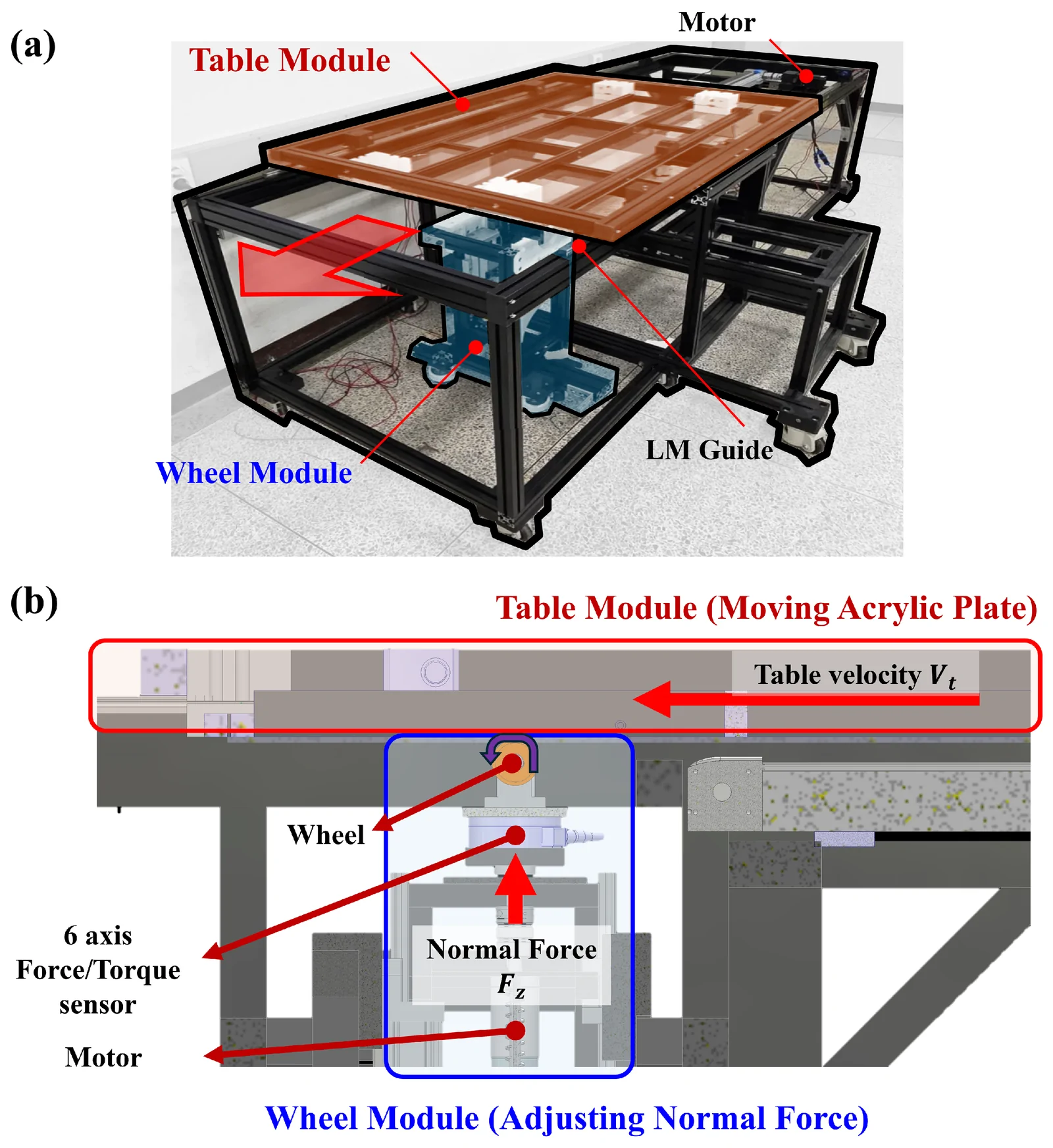
Wheel–wall traction test bench. (**a**) Overview of the test bench for Magic Formula model identification; (**b**) mechanical configuration of the table and wheel modules.

A test bench was developed to measure the longitudinal contact characteristics of the robot’s urethane roller on acrylic for Magic Formula identification (Figure 2).

The test bench consists of a table module that translates an acrylic plate and a wheel module that controls roller rotation and contact loading. The table module uses a ball-screw-driven linear actuator to move the plate at a constant speed, reproducing the relative motion between the roller and the wall surface. The wheel module includes a roller drive motor (XH540-W150-T, DYNAMIXEL, ROBOTIS, Seoul, Republic of Korea), a linear actuator for applying normal loads, a compliant spring mechanism, and a six-axis force/torque sensor (RFT80-6A02, Robotous, Seongnam, Republic of Korea). The normal force and the roller’s angular orientation about the surface-normal axis can be adjusted. The orientation angle was held constant at 0° throughout the experiments. The plate translation speed, roller rotational speed, and normal force can be set independently.

The wheel module uses a urethane roller matching the specifications of the robot’s drive wheels. The longitudinal force $F_{x}$ and normal force $F_{z}$ were measured simultaneously with the six-axis force/torque sensor mounted beneath the wheel module. Compliance in the surface-normal direction was introduced to mitigate contact-load variations caused by local bending of the acrylic plate. Independent control of the roller and plate speeds allows both driving and braking slip conditions associated with ascent and descent to be investigated.

The nominal roller peripheral speed was set to 50 mm/s, while the plate speed was varied to set the slip ratio. The zero-force reference $V_{0}$ was defined as the plate speed at which the measured $F_{x}$ was closest to zero. At 20 N, this reference was $V_{0}=51.3\;\mathrm{mm}/s$. Two separate motor-speed measurements at this load gave a mean angular speed of approximately $\bar{\omega }=2.506\;\mathrm{rad}/s$. The corresponding equivalent rolling radius was $r_{\mathrm{eq},0}=V_{0}/\bar{\omega }\approx 20.47\;\mathrm{mm}$. This estimate characterizes the zero-force speed reference and is distinct from a direct measurement of the loaded wheel radius. The same $V_{0}$ was used to normalize slip at 20 and 30 N.

The experimental slip ratio was defined using the zero-force reference speed $V_{0}$ and plate translation speed $V_{t}$ as

(2) $$\kappa =\frac{V_{0}-V_{t}}{\max\left(\left|V_{0}\right|,\left|V_{t}\right|\right)}.$$

Positive slip (κ>0) represents the driving condition, in which the plate speed is lower than the reference speed. Negative slip (κ<0) represents the braking condition relevant to descent, in which the plate speed exceeds the reference speed.

The target slip ratio ranged from −0.40 to +0.40. To resolve the steep variation in longitudinal force near zero slip, increments of 0.025 were used for |κ|≤0.10, and increments of 0.05 were used elsewhere. An additional near-zero-slip point was measured at 20N, providing 22 data points at 20N and 21 data points at 30N. For each slip-ratio and normal-force condition, the acrylic plate traveled approximately 300 mm. The longitudinal force $F_{x}$ was averaged over the steady-state portion of each run after excluding the transient portion. Each condition was tested five times, and the mean and standard deviation were calculated from the averages of the five runs.

### 3.2. Magic Formula Identification and Slip Prediction for Vertical Motion

**Figure 3:**
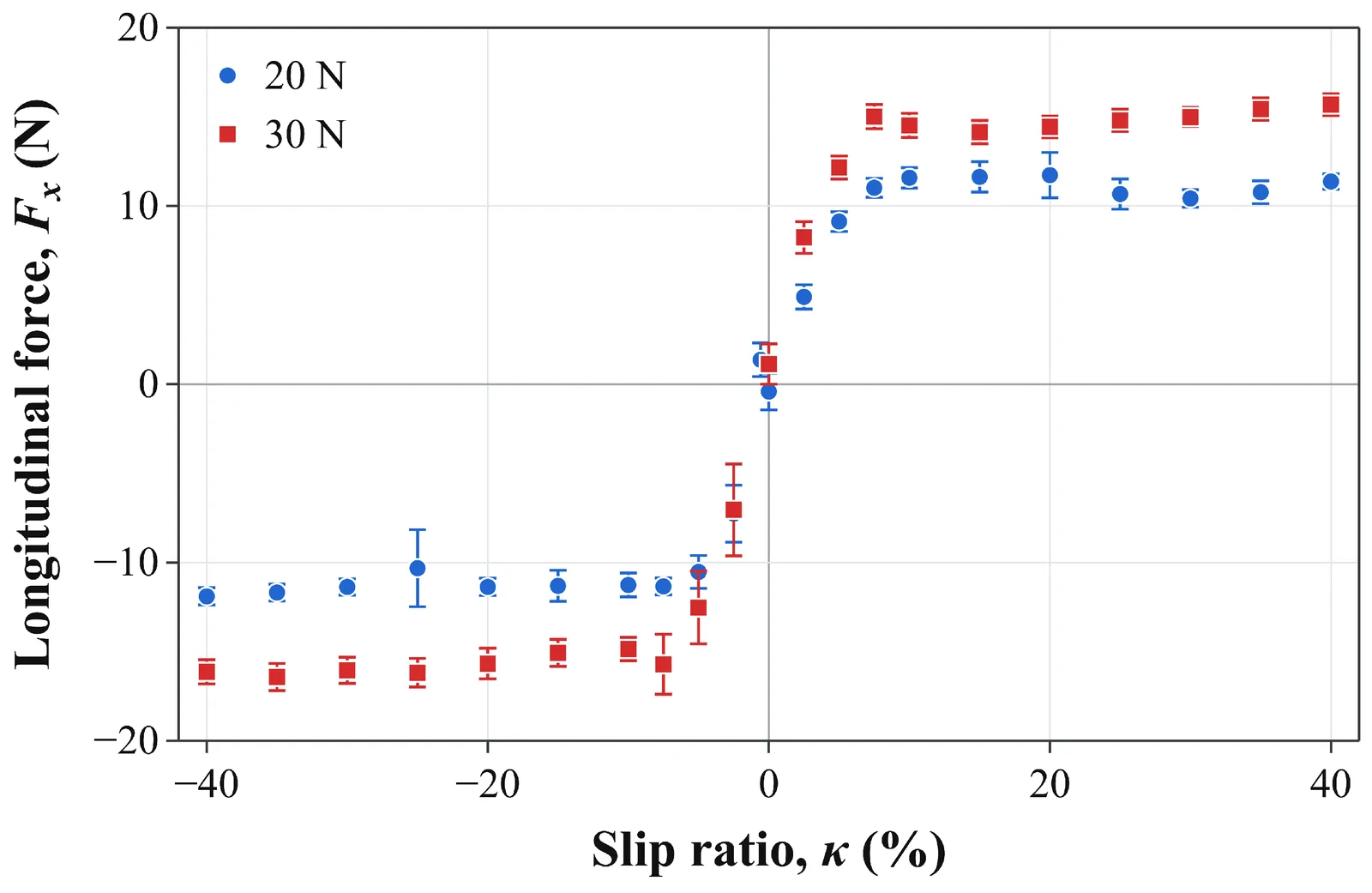
Measured traction–slip data at normal forces of 20 N and 30 N. Markers and error bars show the mean and standard deviation of five run-averaged measurements.

Figure 3 presents the measured traction–slip curves at normal forces of 20 N and 30 N, along with the mean values and standard deviations obtained from five repeated tests. At both loads, the longitudinal force varied rapidly near zero slip. For |κ| above approximately 0.10, the force magnitude approached a plateau or showed a slight decrease. Increasing the normal force increased the peak traction, although the increase was not proportional to the load. The measurements also showed differences between the driving and braking branches. These results indicate that a constant friction coefficient alone cannot adequately characterize the measured traction–slip relationship.

The Magic Formula was selected to represent the steep force variation near zero slip and saturation at larger slip within a compact, smooth model [16]. The longitudinal traction–slip relationship was written as

(3) $$F_{x}\left(\kappa ,F_{z}\right)=D_{x}\sin\;\left[C_{x}\arctan\;\left\{B_{x}\kappa -E_{x}\left(\kappa ,F_{z}\right)\left(B_{x}\kappa -\arctan\left(B_{x}\kappa \right)\right)\right\}\right],$$

where $B_{x}$, $C_{x}$, and $D_{x}$ denote the stiffness, shape, and peak factors, respectively. The curvature factor is defined as

(4) $$E_{x}\left(\kappa ,F_{z}\right)=E_{0x}\left(F_{z}\right)\left[1-q_{x}\mathrm{sgn}\left(\kappa \right)\right],\;q_{x}=0.01444.$$

No horizontal or vertical shift terms were applied. The sign-dependent curvature factor allows the driving and braking branches to differ, while the force remains zero at zero slip.

**Figure 4:**
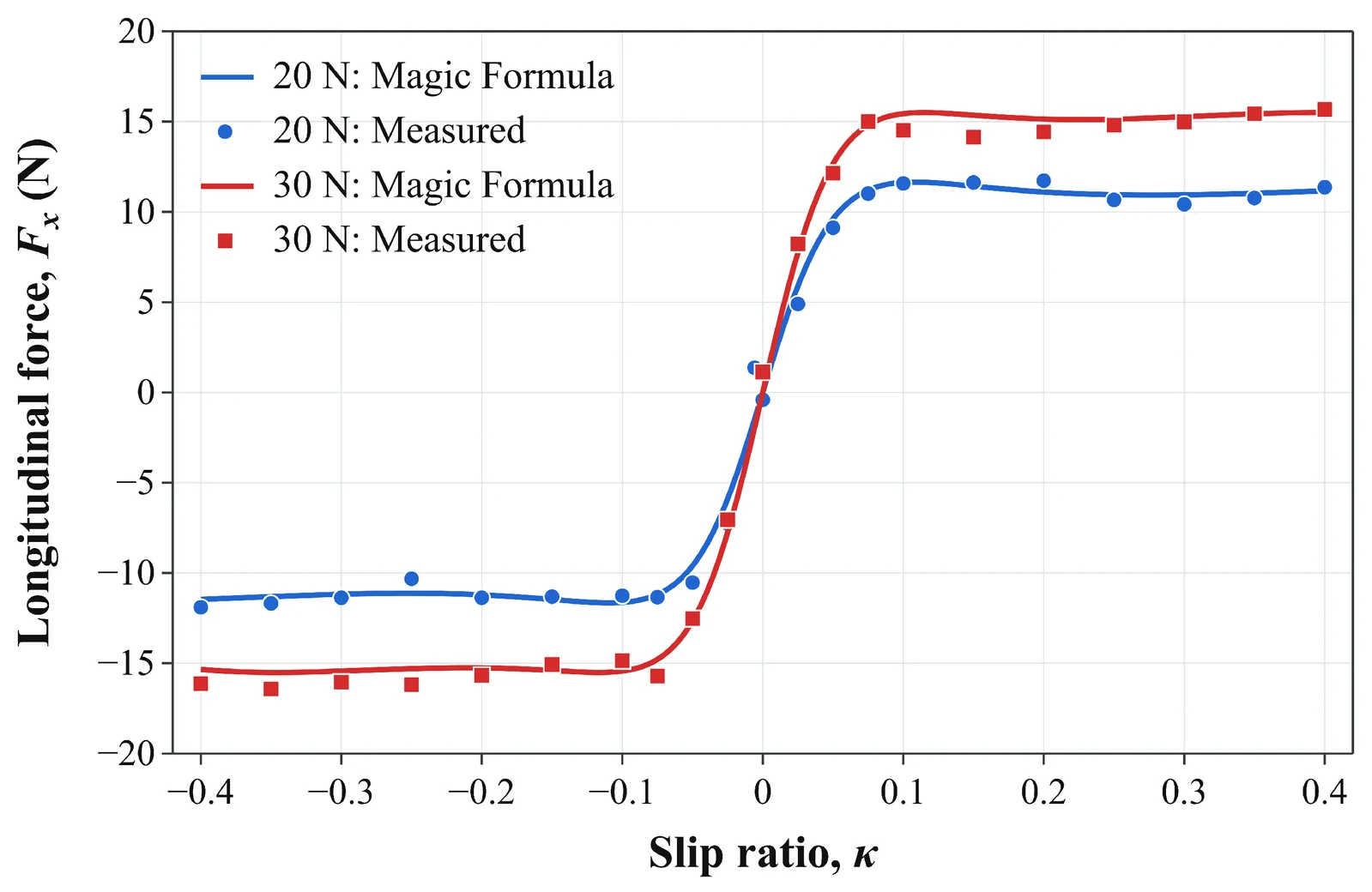
Measured traction–slip data (symbols) and fitted Magic Formula curves (solid lines) at normal forces of 20 N and 30 N.

Table 2a lists the coefficients used to calculate the experimental slip settings. The model uses common values of $C_{x}=2.5$ and $q_{x}=0.01444$, with load-specific values of $B_{x}$, $D_{x}$, and $E_{0x}$. Sharing $C_{x}$ and $q_{x}$ reduces the number of coefficients from ten to eight while retaining load-specific stiffness, peak, and curvature factors. Figure 4 compares the model with the measurements. Using the coefficients before rounding, the fitting RMSEs were 0.810N for the 22 mean-force measurements at 20N and 0.672N for the 21 measurements at 30N. Each mean force received equal weight in the RMSE calculation.

Prediction validation withheld each of the 20 interior slip values in turn, removing all measurements at that slip value from both load datasets. The endpoint measurements remained in the training data. All eight coefficients, including the shared $C_{x}$ and $q_{x}$, were re-estimated by minimizing the sum of squared force residuals over the remaining measurements. Piecewise-linear interpolation used the same training data separately at each load. This procedure yielded 39 predictions at withheld slip values.

The validation fits used the trust-region reflective solver in SciPy 1.18.1 with Jacobian scaling. The parameter bounds were $0.5\leq C_{x}\leq 3$, $-0.5\leq q_{x}\leq 0.5$, $0.1\leq B_{x}\leq 100$, $1\leq D_{x}\leq 40\;N$, and $-3\leq E_{0x}\leq 3$. Each fit used 64 initial parameter sets. Four sets used $C_{x}=1$, 1.5, 2, or 2.5, with $q_{x}=0$, $B_{x}=10$, $E_{0x}=0.5$, and $D_{x}=P_{z}$, where $P_{z}$ is the largest absolute training force at each load. The other 60 sets used uniform sampling over $C_{x}\in \left[0.6,\;2.9\right]$, $q_{x}\in \left[-0.2,\;0.2\right]$, $E_{0x}\in \left[-1,\;2\right]$, and $D_{x}/P_{z}\in \left[0.8,\;1.5\right]$, with logarithmically uniform sampling of $B_{x}\in \left[0.5,\;40\right]$. Sampling used NumPy’s default_rng with seed 261,101. The function, step, and gradient tolerances were $10^{-10}$, with a maximum of 5000 function evaluations per initialization. The solution with the lowest training sum of squared residuals was selected. In Table 2b, the Magic Formula had lower pooled RMSE and lower RMSE at 20N. Piecewise-linear interpolation had lower RMSE at 30N and lower pooled mean absolute error.

The identified model was used to select a representative slip ratio based on the traction required for steady ascent. A nominal equivalent resistance of $F_{B}=1.4\;N$ was adopted before the travel experiments to select the representative slip magnitudes and was kept fixed. This parameter represents the combined resistance of the drive and passive modules.

Three trial records were used for the resistance analysis. With the drive wheels removed, the coupled modules, with a total moving mass of M=2.05kg, descended along the wall under gravity at a varying speed. Position was obtained by image-based tracking. The downward displacement *d* was evaluated over T=0.2s from the first detected downward-motion frame. Assuming zero initial velocity and constant resistance over this interval, the equivalent resistance was estimated as

$$F_{\mathrm{res}}=M\left(g-\frac{2d}{T^{2}}\right),$$

where $g=9.81\;m/s^{2}$. The mean and sample standard deviation were 1.475N and 0.968N, respectively. At the adopted image scale of 1.781mm/pixel, an assumed displacement error of ±1 pixel corresponds to ±0.183N in each resistance estimate.

Removing the drive wheels transfers their normal load to the remaining supports on the drive module. At a fixed magnetic gap and under quasi-static normal equilibrium, the total drive-side ball-plunger load is approximately 82.8N without the wheels, compared with 42.8N and 22.8N at per-wheel normal forces of 20N and 30N, respectively. The passive-module support load remains approximately 82.8N, providing a common resistance contribution under a Coulomb approximation with unchanged contact properties. The total resistance may nevertheless vary as the drive-side loading changes. Accordingly, $F_{B}$ represents a common nominal resistance rather than a load-specific measurement of the assembled robot. Taking the upward direction as positive, the force balance during ascent is

(5) $$M\dot{v}=F_{x,L}+F_{x,R}-Mg-F_{B},$$

where $F_{x,L}$ and $F_{x,R}$ are the upward traction forces exerted on the robot at the left and right drive wheels, respectively. For steady ascent, the total required traction is

(6) $$F_{\mathrm{req}}=Mg+F_{B},\;F_{B}=1.4\;N.$$

Assuming equal traction sharing between the two drive wheels, the representative slip magnitude $\kappa^{*}$ was obtained by solving

(7) $$F_{x}\left(\kappa^{*},F_{z}\right)=\frac{F_{\mathrm{req}}}{2}$$

on the increasing branch of the traction–slip curve near zero slip. The solutions were rounded to obtain the fixed slip magnitudes $\kappa^{*}=0.065$ at 20N and $\kappa^{*}=0.038$ at 30N. Using the coefficients before rounding for presentation in Table 2a, the applied slip magnitudes give predicted traction forces per wheel of 10.761N at $F_{z}=20\;N$ and 10.713N at $F_{z}=30\;N$. These values differ from the nominal steady-ascent requirement of $(Mg+F_{B})/2=10.755\;N$ by +0.06% and −0.39%, respectively. The small differences result from rounding the calculated slip setpoints. These ascent-based slip magnitudes were also used for descent. The signed slip values used in the controller were

(8) $$\kappa_{C}=\kappa^{*},\;\kappa_{D}=-\kappa^{*},$$

where the positive and negative signs denote driving during ascent and braking during descent, respectively. The Magic Formula links the measured traction–slip relationship to the required traction to select the slip operating point at each tested normal force. The resulting slip values were fixed before the travel experiments and used for velocity compensation without adjustment based on the travel results.

The corresponding steady-descent requirement is $(Mg-F_{B})/2\approx 9.355\;N$ per wheel. Using the fitted coefficients before rounding, solving $\left|F_{x}\left(\kappa_{D},F_{z}\right)\right|=\left(Mg-F_{B}\right)/2$ on the braking branch gives $\kappa_{D}\approx -0.04763$ at 20N and $\kappa_{D}\approx -0.03159$ at 30N. At the applied descent settings of −0.065 and −0.038, the predicted braking-force magnitudes per wheel are 10.745 and 10.702N, respectively, exceeding the nominal descent requirement by 14.9% and 14.4%. The ascent-based magnitudes were retained as common nominal settings to evaluate one preset value per normal force in both travel directions. These settings do not enforce exact descent force balance, and their compensation performance is evaluated in Section 5.2.

## 4. Model-Based Feedforward and Path-Tracking Control

### 4.1. Direction-Dependent Feedforward Velocity Compensation

During vertical ascent, the drive wheels provide traction to overcome gravity, whereas braking traction is necessary to maintain a constant descent speed. Under longitudinal slip, the wheel peripheral speed may exceed the robot’s translational speed during ascent, whereas the robot’s translational speed may exceed the wheel peripheral speed during descent. The feedforward command therefore increases the commanded wheel speed during ascent and reduces its magnitude during descent.

The slip values selected in Section 3.2 were used as fixed dimensionless feedforward design parameters. The same magnitude $\kappa^{*}$ was applied in both directions, with $\kappa_{C}=\kappa^{*}$ for ascent and $\kappa_{D}=-\kappa^{*}$ for descent. The reference $V_{0}$ was used to normalize the test-bench slip data for model identification. Robot commands used the resulting slip settings and the nominal wheel radius of 20mm. Neither $V_{0}$ nor an equivalent zero-force calibration was applied as an additional command correction.

Let $v_{r}>0$ denote the target robot speed and $u_{w}>0$ the magnitude of the nominal wheel peripheral-speed command. For command design, the normalization form in Equation (2) was applied as

(9) $$\kappa_{C}=\frac{u_{w}-v_{r}}{u_{w}},\;\kappa_{D}=\frac{u_{w}-v_{r}}{v_{r}}.$$

Taking ascent as the positive wheel-velocity direction, the feedforward commands are

(10) $$v_{w,C}^{\mathrm{ff}}=\frac{v_{r}}{1-\kappa_{C}},\;v_{w,D}^{\mathrm{ff}}=-v_{r}\left(1+\kappa_{D}\right).$$

The slip-ratio sign distinguishes driving from braking, whereas the wheel-velocity sign specifies the direction of robot motion.

Table 3 lists the nominal wheel-velocity commands for the selected slip magnitudes.

The feedforward commands were calculated using the fixed slip values without online slip estimation. In the integrated controller, velocity feedback was incorporated to compensate for residual velocity errors, as detailed in Section 4.2.

### 4.2. Integrated Velocity and Path-Tracking Control

**Figure 5:**
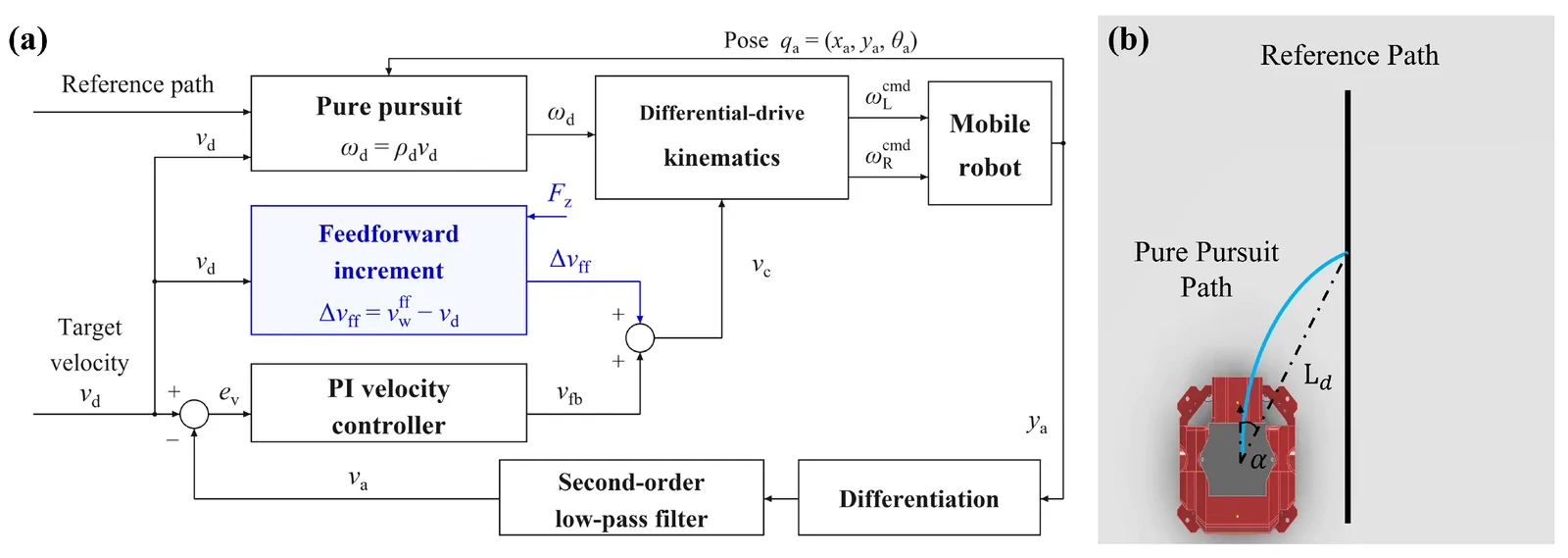
Integrated velocity and path-tracking control. (**a**) Control structure combining Magic Formula feedforward compensation, PI velocity feedback, and pure pursuit path tracking; (**b**) pure pursuit geometry.

The integrated controller combines Magic Formula feedforward compensation, proportional–integral (PI) velocity feedback, and pure pursuit path tracking [22]. Figure 5 shows the control structure and the pure pursuit geometry.

The vertical velocity was estimated by differentiating the vision-based vertical position $y_{a}$. A second-order Butterworth low-pass filter was applied to the differentiated signal to attenuate high-frequency noise. Its continuous-time representation is

(11) $$H\left(s\right)=\frac{\omega_{c}^{2}}{s^{2}+2\zeta \omega_{c}s+\omega_{c}^{2}},$$

where $\omega_{c}=2\pi f_{c}$, ζ=0.707, and $f_{c}=1$ Hz. The filtered vertical velocity, denoted as $v_{a}$, was used to evaluate the feedforward experiments and as the velocity feedback signal.

The signed target velocity is $v_{d}=+v_{r}$ for ascent and $v_{d}=-v_{r}$ for descent. The velocity error, integral state, and PI output are given by

(12) $$\begin{array}{cc} e_{v}\left[k\right] & =v_{d}\left[k\right]-v_{a}\left[k\right],\\ I[0] & =0,\\ I[k] & =I\left[k-1\right]+\Delta t_{k}e_{v}\left[k\right],\;k\geq 1,\\ v_{\mathrm{fb}}\left[k\right] & =K_{pv}e_{v}\left[k\right]+c_{I}I\left[k\right], \end{array}$$

where $K_{pv}=0.8$, $c_{I}$ is the integral-state coefficient, and $\Delta t_{k}=t_{k}-t_{k-1}$ is the actual elapsed time between consecutive control updates. For each ascent or descent segment, k=0 denotes the first control update, at which the integral state is set to zero. From k=1 onward, the state accumulates the current velocity error multiplied by the actual elapsed time. The integral state is reset to zero whenever the controller transitions from a stationary state to a new motion segment.

In the integrated controller, the feedforward increment was added to the PI output:

(13) $$\begin{array}{cc} \Delta v_{\mathrm{ff}} & =v_{w}^{\mathrm{ff}}-v_{d},\\ v_{c} & =v_{\mathrm{fb}}+\Delta v_{\mathrm{ff}}, \end{array}$$

where $v_{w}^{\mathrm{ff}}$ is defined by Equation (10) and $v_{c}$ is the nominal mean wheel peripheral-velocity command. Disabling feedforward sets $\Delta v_{\mathrm{ff}}=0$. The controller gains and system parameters are summarized in Table 4.

Pure pursuit was employed for lateral path tracking. A target point was selected along the reference path in the intended direction of travel. Let $L_{d}$ denote the straight-line distance from the robot’s reference point to the target point, and let α denote the signed angle measured from the robot’s body-forward direction to the line extending from its reference point to the target point. The commanded curvature and angular velocity are

(14) $$\rho_{d}=\frac{2\sin\alpha }{L_{d}},\;\omega_{d}=\rho_{d}v_{d}.$$

For descent, the reference path order was reversed. The angle α remains referenced to the body-forward direction, while $v_{d}$ is negative.

The mean wheel velocity command $v_{c}$ and angular velocity command $\omega_{d}$ are converted into individual wheel commands using differential-drive kinematics,

(15) $$\begin{array}{cc} v_{R} & =v_{c}+\frac{b}{2}\omega_{d},\\ v_{L} & =v_{c}-\frac{b}{2}\omega_{d},\\ \omega_{R}^{\mathrm{cmd}} & =\frac{v_{R}}{r},\;\omega_{L}^{\mathrm{cmd}}=\frac{v_{L}}{r}, \end{array}$$

where *b* is the distance between the left and right wheel centers and *r* is the wheel radius. The control contribution is the integration of the experimentally identified traction–slip characteristics into the wheel velocity commands for ascent and descent within the existing path-tracking structure.

## 5. Experimental Validation and Discussion

### 5.1. Experimental Setup and Evaluation Protocol

**Figure 6:**
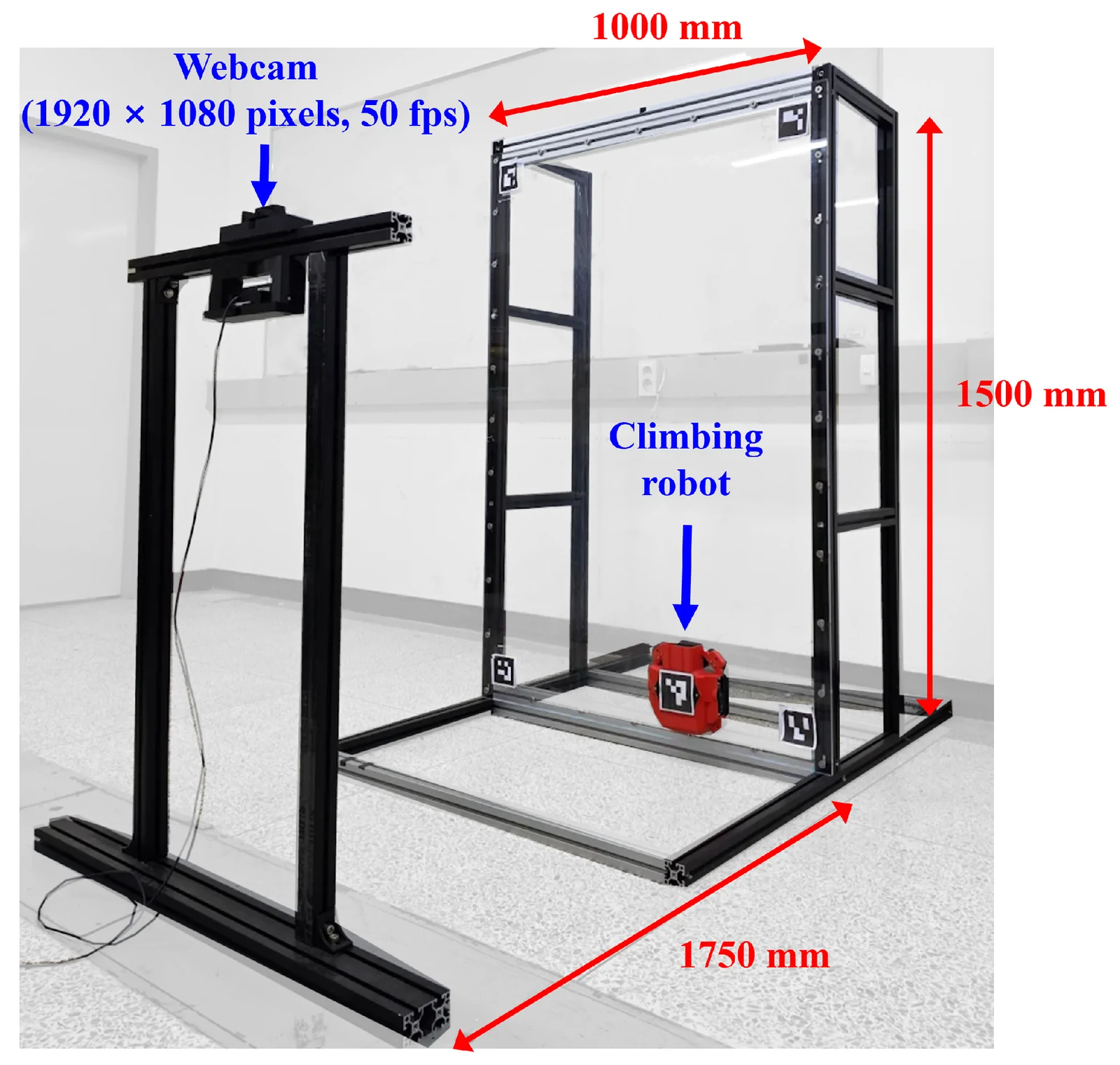
Experimental setup. Vertical acrylic panel and vision-based measurement arrangement.

Experiments were conducted on a vertical acrylic panel with a width of 1000 mm, a height of 1500 mm, and a thickness of 8 mm (Figure 6). An external camera was positioned approximately 1.75m from the panel. Four reference markers affixed to the panel established the wall coordinate system, while a marker mounted on the robot enabled estimation of its position and orientation. The image resolution was 1920×1080 pixels, with nominal horizontal and vertical fields of view of 88.4° and 57.6°, respectively. At a camera-to-marker-plane distance of approximately 1.75m, a plane perpendicular to the optical axis gives approximate image scales of 1.77mm/pixel horizontally and 1.78mm/pixel vertically.

Variation in the vision outputs was assessed using commanded holds in 48 travel recordings. The interval from 1.0 to 1.9s after the first sample of each hold was analyzed when both motor commands and measured wheel velocities were zero. For 78 upper and 58 lower holds, the pooled within-hold standard deviations were 0.37 and 0.50mm in *x*, 1.91 and 4.46mm in *y*, and 0.57 and 0.77mm/s in the filtered velocity, respectively. These signal variations may include residual motion, filter transients, and repeated position samples. Absolute position and velocity errors were not established against an independent reference. The reported tracking errors are referenced to the same external vision estimates used for feedback. Motor commands generated on the external PC were transmitted to the ESP32 controller via Wi-Fi. The system and control parameters are summarized in Table 4.

The integral-state coefficient $c_{I}$ was 2.0 at 20N and 80mm/s, and 2.7 at both 30N conditions. At 20N and 100mm/s, $c_{I}$ was 2.3 in all PI recordings and four recordings with PI control and feedforward compensation, and 2.7 in the remaining two recordings with PI control and feedforward compensation. The comparison of PI control with and without feedforward compensation used the recordings with matching $c_{I}=2.3$ at this condition. Experiments were conducted in two stages, both illustrated in Supplementary Video S1. In the first stage, the feedforward compensation was evaluated without longitudinal position or velocity feedback. Precomputed wheel-speed commands were applied, and a separate PI controller suppressed the yaw rotation. Therefore, the experiments were open-loop with respect to longitudinal motion. Vision-based position measurements were used to evaluate travel distance and velocity.

The normal force was set to either 20 or 30N per wheel, and the target speed to either 80 or 100mm/s. The nominal travel distance was 800mm, corresponding to motion command durations of 10s and 8s, respectively. Five recordings were collected for each combination of normal force, target speed, and compensation setting. Each recording comprised two ascent–descent cycles, yielding ten segments in each travel direction.

Mean speeds in Table 5 were calculated after excluding the first 2s of each segment to reduce the influence of startup transients. For each travel direction, the two segment mean speeds were averaged within each recording. The mean and sample standard deviation were then calculated across the five recordings.

Travel distance was measured from the interpolated position at t=2s to the final position of the segment. The reference distances were 640mm at 80mm/s and 600mm at 100mm/s. The absolute travel-distance error was calculated for each segment and then averaged over the ten segments in each travel direction.

The second stage assessed the performance of the integrated controller, which combined feedforward compensation, PI velocity feedback, and pure pursuit path tracking. The reference path was a vertical straight line. The first ascent began at y=0 and terminated at y=1.2m. The robot subsequently descended to y=0.1m, after which successive segments alternated between y=0.1m and y=1.2m. For each combination of normal force and target speed, six ascent–descent cycles were completed from each of two initial lateral offsets of approximately +0.1m and −0.1m. This yielded a total of 12 ascent segments and 12 descent segments for each test condition.

An additional comparison assessed the effect of feedforward with PI feedback and pure pursuit active in both cases. The comparison matched the PI coefficients, the reference line x=−0.4m, and the look-ahead distance of 0.15m. For each normal force, target speed, and controller, the earliest two eligible recordings from each initial lateral offset of approximately −0.1m and +0.1m were selected. Each recording contained two ascent–descent cycles, giving four recordings and eight segments per direction.

Table 6 and Table 7 summarize the full dataset for PI control with feedforward compensation, comprising six recordings and 12 segments per direction for each normal-force and target-speed condition. The additional comparison uses the selected four recordings per controller and condition.

Velocity and lateral tracking performance were evaluated using

(16) $${\mathrm{RMSE}}_{v}=\sqrt{\frac{1}{N_{v}}\sum_{k=1}^{N_{v}}{\left[v_{d}\left(t_{k}\right)-v_{a}\left(t_{k}\right)\right]}^{2}},$$

and

(17) $${\mathrm{RMSE}}_{x}=\sqrt{\frac{1}{N_{x}}\sum_{k=1}^{N_{x}}{\left[x_{a}\left(t_{k}\right)-x_{\mathrm{ref}}\left(t_{k}\right)\right]}^{2}},$$

where $v_{d}$ is the signed target velocity, $v_{a}$ is the filtered vision-based vertical velocity, $x_{a}$ is the measured lateral position, and $x_{\mathrm{ref}}$ is the lateral coordinate of the reference path. The velocity signal was processed using the filter described in Section 4.2. The sample counts $N_{v}$ and $N_{x}$ correspond to the velocity and lateral-position evaluation intervals, respectively.

Time was measured from the first sample of each motion segment. The mean speeds and RMSE values in Table 6 were calculated after excluding the first 2s. Velocity RMSE over the same interval was also calculated for the comparison of PI control with and without feedforward compensation. The same filtered velocity signal was used for the velocity plots and performance metrics. Mean speeds are reported as magnitudes.

For Table 6, each metric was calculated separately for each segment and averaged across the 12 segments in each travel direction. For the comparison of PI control with and without feedforward compensation, the two segment metrics for each direction were first averaged within each recording. The mean and sample standard deviation were then calculated across the four recordings.

Aggregate error reductions were evaluated using a stratified cluster bootstrap with 100,000 resamples. Whole recordings, including all their segments, were resampled within each normal-force, target-speed, and controller group. The matched PI comparison was additionally stratified by initial lateral-offset side. Controller groups were resampled independently, and the eight operating conditions retained equal weights. For an aggregate error *E*, the relative reduction was $100(1-E_{\mathrm{with}}/E_{\mathrm{without}})$, where the subscripts indicate whether feedforward compensation was used. The 95% confidence intervals are the 2.5th and 97.5th percentiles of the bootstrap estimates and describe between-recording variability under the tested conditions.

Full-segment velocity metrics included the first 2s and extended from the first to the last sample of each ascent or descent phase, excluding the subsequent stationary hold. Startup velocity RMSE was also calculated over the first 2s for the experiments without longitudinal feedback. RMSE was calculated separately for each segment and then averaged.

Peak overshoot was defined as the largest positive excess of velocity in the intended travel direction over the target speed, or zero if no excess occurred. Table 7 reports the mean segment RMSE, mean peak overshoot, and maximum segment peak for the integrated controller.

Settling time was defined as the earliest sample time from which $\left|v_{a}-v_{d}\right|\leq 0.05\left|v_{d}\right|$ held until the segment ended, with at least 0.5s of observations remaining. The number of segments meeting this criterion and their median settling time are reported. Segments that did not meet the criterion were not assigned a finite settling time.

The first ascent from each prescribed initial offset included convergence toward the reference path. Subsequent segments started from the position reached at the end of the preceding segment. Therefore, the prescribed lateral offset did not apply to every ascent. Descent segments began after the robot had converged toward the reference path. These differences in initial conditions are considered in the interpretation of the results in Section 5.3.

### 5.2. Feedforward Compensation Results

**Figure 7:**
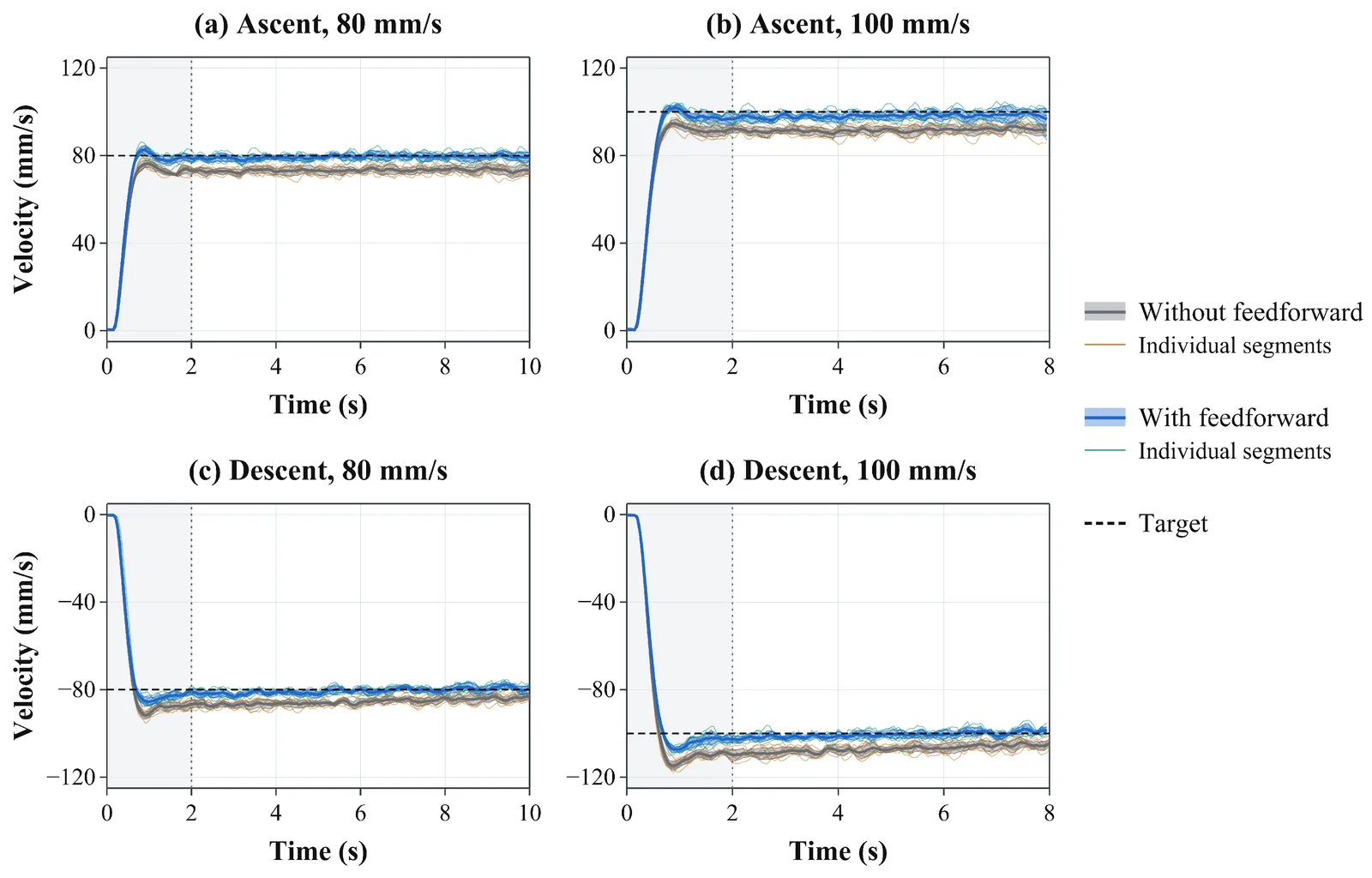
Velocity responses with and without feedforward compensation at 20 N per wheel: (**a**,**b**) ascent and (**c**,**d**) descent, at 80 mm/s (**left**) and 100 mm/s (**right**). Faint and thick curves show individual segments and their mean; colored bands indicate ± one standard deviation (n=10). The gray background marks the first 2s of each motion segment.

**Figure 8:**
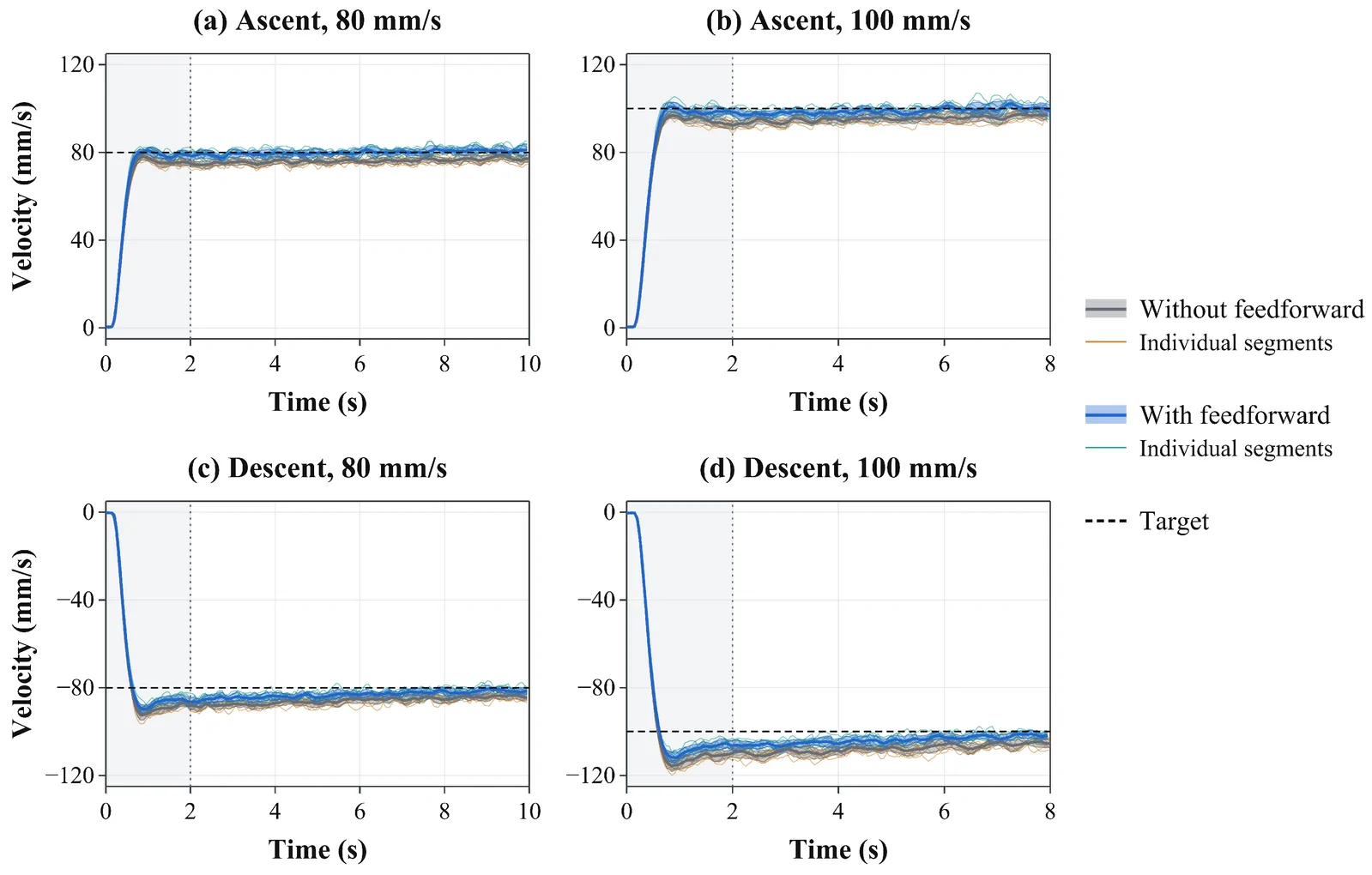
Velocity responses with and without feedforward compensation at 30N per wheel: (**a**,**b**) ascent and (**c**,**d**) descent, at 80mm/s (**left**) and 100mm/s (**right**). Faint and thick curves show individual segments and their mean; colored bands indicate ± one standard deviation (n=10). The gray background marks the first 2s of each motion segment.

**Figure 9:**
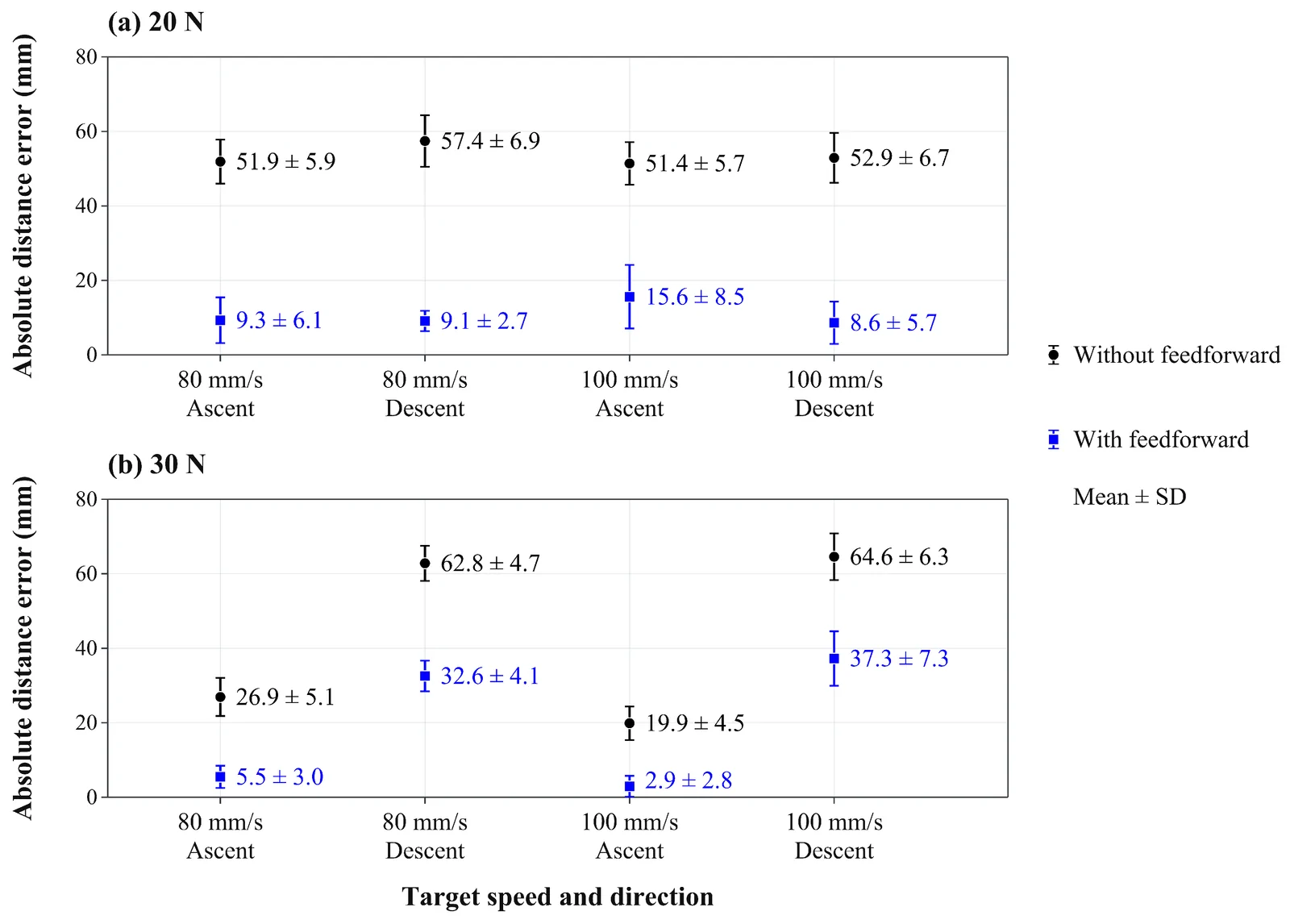
Absolute travel-distance errors with and without feedforward compensation at normal forces of (**a**) 20 N and (**b**) 30 N per wheel. Markers and error bars show the mean and standard deviation of ten segments from five recordings per condition.

Figure 7 and Figure 8 show the velocity responses with and without the feedforward compensation at normal forces of 20 and 30N per wheel, respectively. Table 5 summarizes the mean speeds after excluding the first 2s of each segment. Descent speeds in Table 5 are reported as magnitudes, whereas descent velocities in Figure 7 and Figure 8 are negative. Figure 9 shows a comparison of the absolute travel-distance errors across the tested conditions. Each condition included ten segments per travel direction from five recordings, as described in Section 5.1.

The baseline and feedforward control commands were evaluated in the absence of longitudinal position or velocity feedback. Under baseline conditions, the mean ascent speed was below the target, whereas the mean descent speed exceeded it. Feedforward compensation increased the wheel-speed command during ascent and decreased the magnitude of the descent command. These adjustments reduced the deviation of the mean speed from the target across all eight operating conditions.

For each condition, the absolute difference between the mean and target speeds was calculated before rounding. Averaged equally across the eight conditions, this error decreased from 6.22 to 1.50mm/s, a reduction of 75.9% with a 95% confidence interval of 74.2–77.3%. The mean absolute travel-distance error decreased from 48.5 to 15.1mm, a reduction of 68.8% with a 95% confidence interval of 67.0–70.8%. Table 8 summarizes these results and the velocity RMSE comparisons.

Velocity RMSE decreased by 60.0% after the first 2s and by 5.5% over the full motion segments (Table 8). Startup RMSE decreased from 37.53 to 36.95mm/s. Mean descent peak overshoot decreased at all four load–speed conditions. With compensation, mean ascent peak overshoot ranged from 3.68 to 4.91mm/s across conditions. Across 20 recordings per controller, the settling criterion was met in 11 of 80 segments without compensation and 60 of 80 segments with compensation.

Residual errors were largest during descent at 30N, with compensated mean speeds of 83.1 and 103.9mm/s for targets of 80 and 100mm/s, respectively. These results assess the common ascent-based settings used in the travel experiments.

The model identified at a nominal roller peripheral speed of 50mm/s provided the fixed slip settings used at target speeds of 80 and 100mm/s without retuning. With equal weighting across the two loads and both travel directions, the post-2s mean-speed errors decreased by 79.6% and 72.9% at these target speeds. The corresponding mean absolute travel-distance errors decreased by 71.6% and 65.9%. The model has no explicit speed term, although viscoelastic deformation and interfacial friction can make contact forces speed dependent [23]. These results support the compensation at the tested speeds but do not establish that the full traction–slip relationship is independent of speed.

### 5.3. Integrated Path- and Velocity-Tracking Results and Discussion

**Figure 10:**
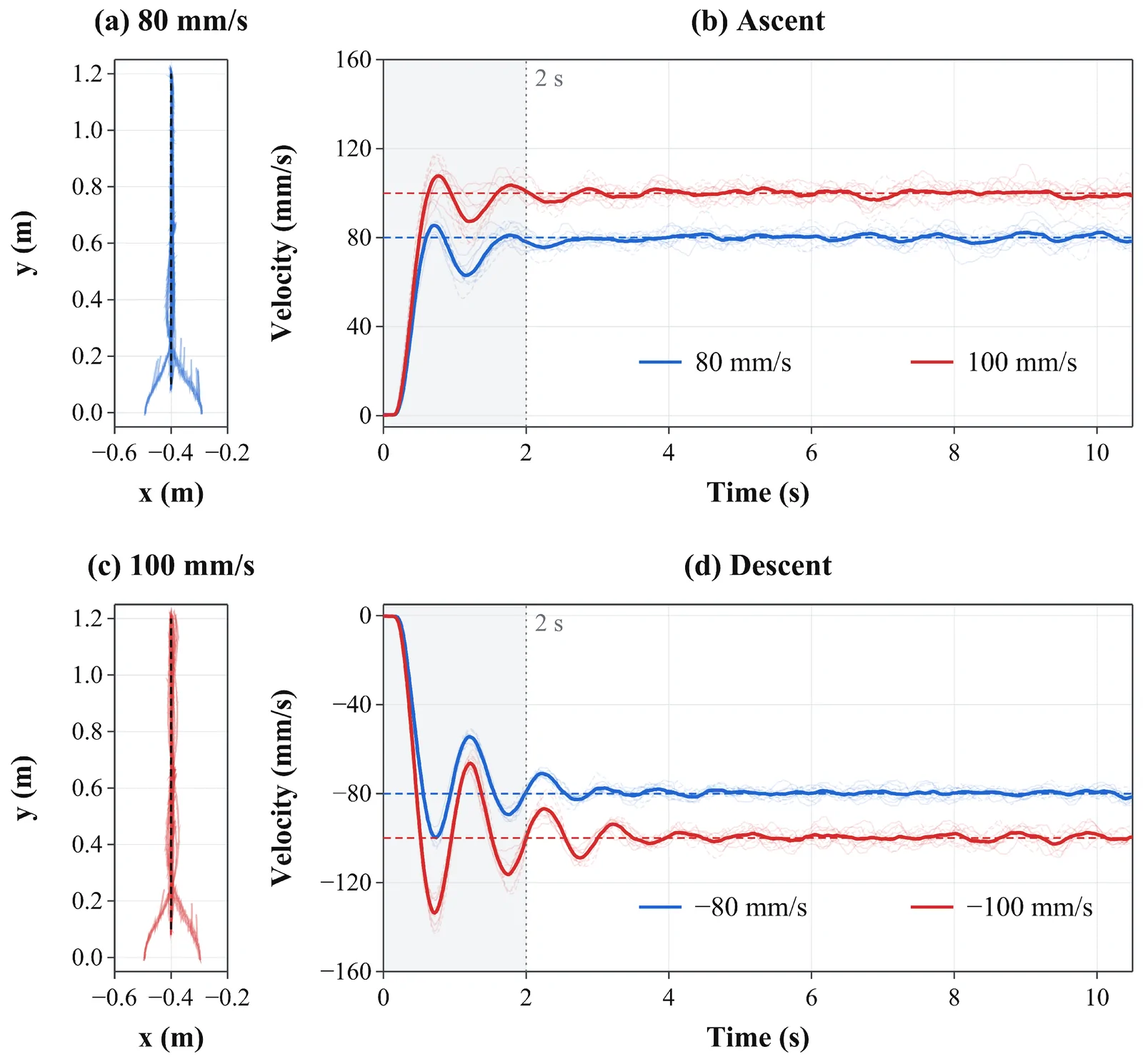
Integrated path and velocity tracking at 20 N per wheel: (**a**,**c**) trajectories at 80 and 100 mm/s, respectively; (**b**) ascent and (**d**) descent velocity responses at both speeds. In (**b**,**d**), faint and thick curves represent individual segments and their mean, respectively (n=12). The gray background marks the first 2s of each motion segment.

**Figure 11:**
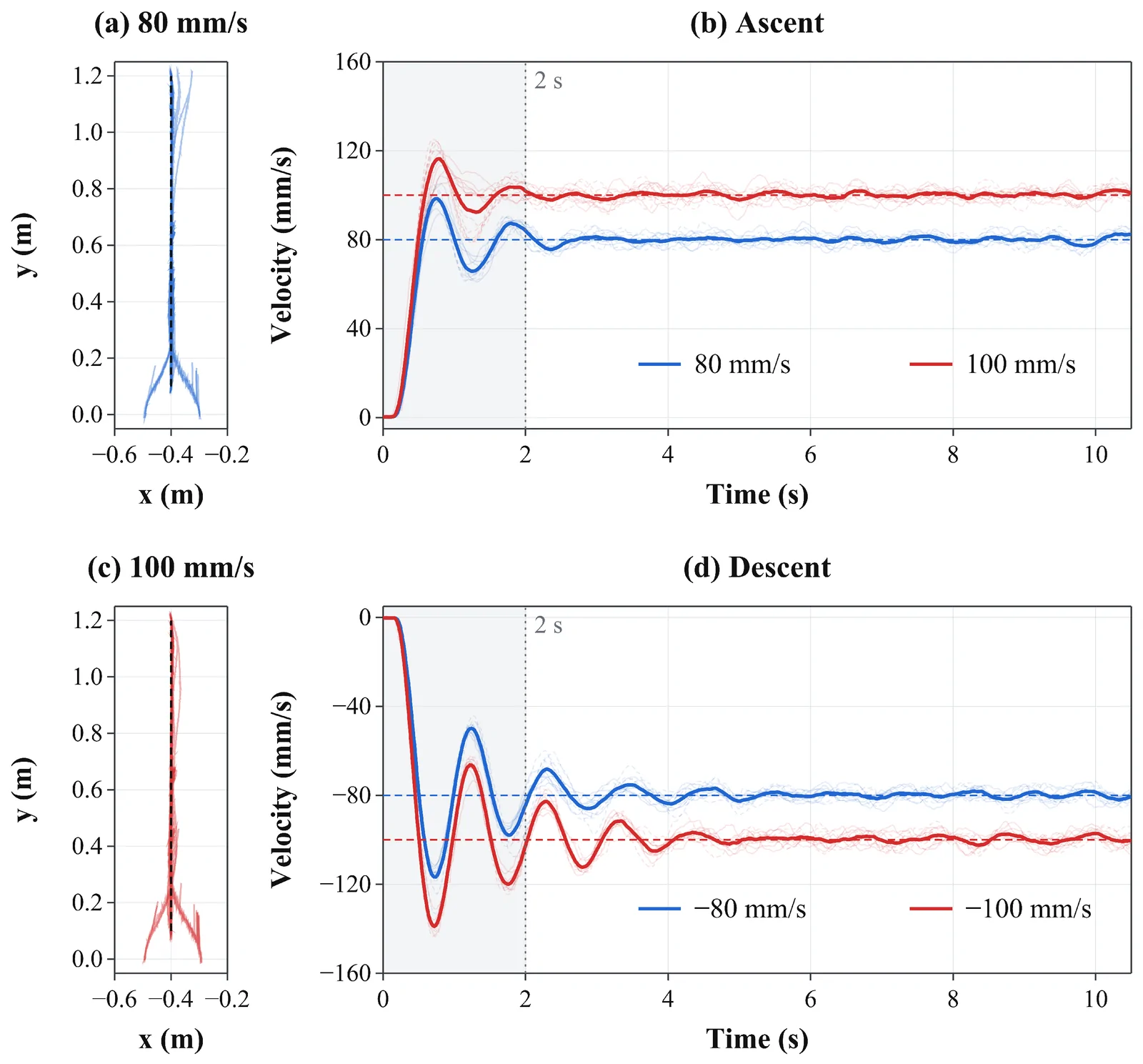
Integrated path and velocity tracking at 30 N per wheel: (**a**,**c**) trajectories at 80 and 100 mm/s, respectively; (**b**) ascent and (**d**) descent velocity responses at both speeds. In (**b**,**d**), faint and thick curves represent individual segments and their mean, respectively (n=12). The gray background marks the first 2s of each motion segment.

The integrated experiments employed Magic Formula-based feedforward compensation, PI velocity feedback, and pure pursuit path tracking. Table 6 summarizes the results, and Figure 10 and Figure 11 present the trajectories and velocity responses under normal forces of 20 and 30N per wheel, respectively. Each table entry represents the arithmetic mean of the segment-level metric across 12 segments for the specified direction of travel, as described in Section 5.1. Descent speeds in Table 6 are expressed as magnitudes, whereas the descent velocities plotted in Figure 10 and Figure 11 are negative.

After excluding the first 2s of each segment, the mean speed was close to the target in all conditions. The condition-level velocity RMSE ranged from 2.61 to 4.93mm/s, yielding an arithmetic mean of 3.70mm/s across the eight conditions. Over the same interval, the mean lateral RMSE across the eight conditions was 5.0mm. The robot converged toward the reference path from the specified initial lateral offsets of approximately ±0.1m. Path divergence and loss of wall attachment were not observed in any of the experimental runs. These results demonstrate that the feedforward compensation could be combined with velocity feedback and pure pursuit to track the vertical reference path at the tested normal forces and target speeds.

Wall attachment was assessed by visual observation during travel on the acrylic panel at wheel normal forces of 20 and 30N per wheel and target speeds of 80 and 100mm/s. The magnetic attraction of 82.8N is a nominal value at a 16mm magnet-face gap. The reaction estimates in Section 2.2 describe the nominal contact-load distribution. Magnetic-force variation during motion and the detachment threshold were not measured, so a quantitative safety margin against detachment was not established.

The lateral RMSE ranged from 6.7 to 8.7mm during ascent and from 1.6 to 2.6mm during descent. As described in Section 5.1, the first ascent included initial path convergence, whereas descent began after convergence. Table 7 summarizes the full-segment response of the integrated controller. Velocity RMSE ranged from 12.98 to 19.73mm/s. Peak overshoot occurred within the first 2s in all 48 descent segments.

In the 30N descent logs, the PI integral term initially built up in the descent direction while the measured speed remained low. The command peak was followed by a measured velocity peak and feedback correction. This sequence is consistent with the combined dynamics of the drive system, PI controller, and filtered velocity feedback, which are not explicitly represented by the steady-state feedforward model.

Later excursions outside the ±5% tolerance band delayed settling or prevented the criterion from being met within the interval. The reported settling-time medians therefore apply only to the qualifying segments. The post-2s metrics describe the evaluation interval and do not imply that every segment settled within 2s.

**Figure 12:**
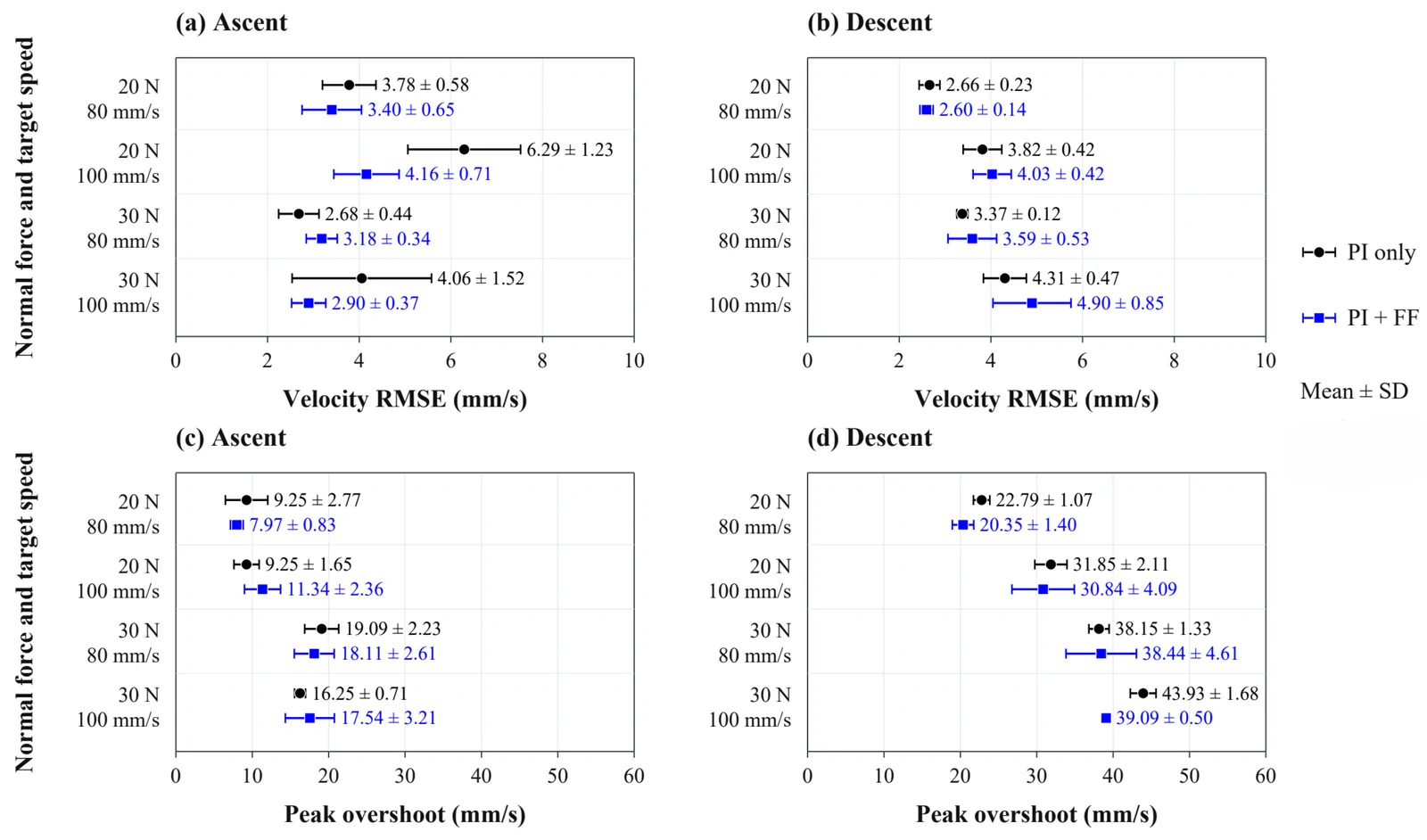
Comparison of PI control with and without feedforward compensation. (**a**,**b**) Velocity RMSE after the first 2s. (**c**,**d**) Peak overshoot over the full segment. Markers and error bars show the mean and sample standard deviation across four recordings for each controller and condition, for ascent (left) and descent (right).

Figure 12 compares PI control with and without feedforward compensation using recordings with matching PI coefficients. Averaged equally across the eight operating conditions, velocity RMSE after the first 2s decreased from 3.87 to 3.59mm/s, a reduction of 7.2% with a 95% confidence interval of 0.5–13.3%.

For the same comparison, mean full-segment RMSE was nearly unchanged at 16.19 and 16.18mm/s without and with feedforward compensation, respectively. The relative reduction was 0.1%, with a 95% confidence interval from −0.9% to 1.1% (Table 8). During ascent, RMSE decreased in three of the four conditions, and the mean decreased from 4.20 to 3.41mm/s, a reduction of 18.9%. During descent, peak overshoot decreased in three conditions, and its mean decreased from 34.18 to 32.18mm/s, a reduction of 5.9%.

PI control alone maintained mean speeds close to the targets. Descent RMSE after the first 2s increased from 3.54 to 3.78mm/s, indicating that the additional effect depended on travel direction and the performance measure.

The results are subject to several limitations. The Magic Formula model was identified under steady-state conditions at two normal forces by using a single urethane–acrylic contact pair. Its applicability to other normal forces, roller hardnesses, or contaminated or wet surfaces requires further validation. The model also does not describe transient slip during acceleration and deceleration.

The position and velocity feedback relied on an external vision system. Deployment beyond the instrumented test environment would require a localization arrangement suited to the target structure. Possible approaches include onboard vision and sensor fusion using inertial measurements, wheel encoders, or ultra-wideband positioning.

## 6. Conclusions

This study presented a model-based feedforward compensation method to reduce velocity errors during the vertical motion of a magnetically coupled wall-climbing robot. Permanent magnets positioned on opposite sides of the wall maintained magnetic coupling, while a spring-preloaded parallel-link mechanism enabled adjustment of the normal forces at the drive wheels under fixed magnetic coupling conditions. This configuration functionally separated magnetic retention from wheel contact-load adjustment.

A dedicated test bench was employed to characterize the longitudinal traction–slip relationships at the urethane roller–acrylic interface under normal forces of 20 and 30 N. Magic Formula models were used to represent the measured relationships. Representative slip magnitudes were calculated from the models and the ascent force balance using a nominal equivalent resistance of 1.4N. The selected slip values of 6.5% and 3.8% were fixed prior to the travel experiments and implemented for both ascent and descent via direction-dependent wheel-velocity commands.

In vertical travel experiments conducted without longitudinal feedback, the mean absolute error of the condition-level mean speed across eight operating conditions decreased from 6.22 to 1.50mm/s, corresponding to a reduction of approximately 75.9%. The mean absolute travel-distance error decreased from 48.5 to 15.1mm, corresponding to a reduction of approximately 68.8%. Repeated experiments integrating feedforward compensation, PI velocity feedback, and pure pursuit path tracking produced velocity and lateral tracking RMSE values, averaged across operating conditions, of 3.70mm/s and 5.0mm, respectively. These metrics were calculated using data collected after the first 2s of each segment. The robot converged toward the reference path from the prescribed initial lateral offsets and completed repeated ascent–descent cycles. These experiments demonstrated that the feedforward method could be incorporated into the existing tracking controller.

In the matched comparison with PI feedback, the additional benefit of feedforward compensation was modest and depended on the evaluation interval and travel direction. After the first 2s of each motion segment were excluded, mean velocity RMSE across the eight operating conditions decreased by 7.2%, with a decrease in mean ascent RMSE and an increase in mean descent RMSE. Full-segment velocity RMSE was nearly unchanged (Table 8).

The findings are limited to the tested normal forces, contact materials, and operating conditions. In addition, the robot requires access to both sides of the wall, which may restrict its applicability in practical environments. Further work should evaluate the system under a broader range of loads, surface conditions, and transient motion, and should investigate onboard localization to enable operation without an external vision system.

## Figures and Tables

**Table 1:** Main design parameters of the wall-climbing robot.

Parameter	Value
Drive-module mass	1.50 kg
Passive-module mass	0.55 kg
Nominal magnetic attraction per module at a 16 mm magnet-face gap	82.8 N
Wheel diameter	40 mm
Urethane roller hardness	50 (Shore A)
Number of driven wheels	2

**Table 2:** Effective model coefficients and prediction validation. (**a**) Coefficients and fitting RMSE of the model used to select the experimental slip settings. (**b**) Prediction errors at withheld interior slip values.

**(a)**						
**$F_{z}$ (N)**	$B_{x}$	$C_{x}$	**$D_{x}$ (N)**	$E_{0x}$	$q_{x}$	**RMSE (N)**
20	8.772	2.500	11.649	1.186	0.01444	0.810
30	8.658	2.500	15.505	1.280	0.01444	0.672
**(b)**						
**$F_{z}$ (N)**		$n_{\mathrm{val}}$	**RMSE (N)**		**MAE (N)**	
			**Magic Formula**	**Linear Interpolation**	**Magic Formula**	**Linear Interpolation**
20		20	1.006	1.333	0.748	0.862
30		19	1.053	0.860	0.942	0.607
Pooled		39	1.029	1.128	0.842	0.738

**Table 3:** Nominal feedforward wheel-velocity commands calculated from the fixed slip values used in the experiments.

$F_{z}$ (N)	$\kappa_{C}$ (%)	$\kappa_{D}$ (%)	$v_{r}$ (mm/s)	$v_{w,C}^{\mathrm{ff}}$ (mm/s)	$v_{w,D}^{\mathrm{ff}}$ (mm/s)
20	6.5	−6.5	80	85.56	−74.80
20	6.5	−6.5	100	106.95	−93.50
30	3.8	−3.8	80	83.16	−76.96
30	3.8	−3.8	100	103.95	−96.20

**Table 4:** System and control parameters.

Category	Parameter	Value
Velocity PI	$K_{pv}/c_{I}$	0.8/condition-dependent
Heading PI	Proportional/integral gains	2.0/0.6
Pure pursuit	Look-ahead distance $L_{d}$	0.15m
Robot geometry	Wheel separation *b*	132mm
	Wheel radius *r*	20mm
Controller	Control rate	50Hz
Camera	Model	STCOM BIZ FHD60F
	Resolution	1920×1080 pixels
	Acquisition frame rate	50fps
ArUco	Marker size	80mm
	Pose-estimation rate	50Hz
Load cell	Model	DBCM-50

**Table 5:** Mean speeds with and without feedforward compensation after excluding the first 2s.

Normal Force (N)	Target Speed (mm/s)	Direction	Without FF (mm/s)	With FF (mm/s)
20	80	Ascent	73.2±0.8	79.3±0.2
20	80	Descent	85.5±0.2	80.6±0.3
20	100	Ascent	91.6±0.4	98.1±0.5
20	100	Descent	107.1±0.6	100.6±0.1
30	80	Ascent	76.1±0.2	79.9±0.3
30	80	Descent	85.8±0.2	83.1±0.4
30	100	Ascent	95.3±0.3	98.9±0.5
30	100	Descent	107.7±0.1	103.9±0.3

Values are means ± sample standard deviations across five recordings. The two segment mean speeds in each travel direction were averaged within each recording before calculating these statistics. Descent speeds are reported as magnitudes. FF denotes feedforward compensation.

**Table 6:** Path- and velocity-tracking results with the integrated controller.

Normal Force (N)	Target Speed (mm/s)	Direction	Mean Speed (mm/s)	Velocity RMSE (mm/s)	Lateral RMSE (mm)
20	80	Ascent	79.6	3.63	8.0
20	80	Descent	79.5	2.61	1.6
20	100	Ascent	98.9	4.82	8.7
20	100	Descent	99.3	4.12	1.9
30	80	Ascent	79.7	2.96	8.7
30	80	Descent	79.7	3.46	2.6
30	100	Ascent	100.0	3.09	6.7
30	100	Descent	99.4	4.93	1.9

**Table 7:** Velocity response metrics over the full motion segments with the integrated controller.

Normal Force (N)	Target Speed (mm/s)	Direction	Full-Segment RMSE (mm/s)	Peak Overshoot Mean [Maximum] (mm/s)	Settled Segments	Settling Time, Median (s)
20	80	Ascent	13.06	8.93 [16.09]	3/12	13.24
20	80	Descent	13.79	19.89 [25.06]	8/12	12.03
20	100	Ascent	17.79	11.74 [17.45]	3/12	10.46
20	100	Descent	19.32	33.77 [42.94]	9/12	9.58
30	80	Ascent	12.98	19.08 [25.31]	4/12	13.89
30	80	Descent	15.11	36.99 [53.37]	4/12	12.86
30	100	Ascent	17.60	18.16 [25.21]	2/12	10.51
30	100	Descent	19.73	39.12 [43.47]	9/12	9.78

**Table 8:** Effect of feedforward compensation and 95% confidence intervals for the relative reductions.

Metric	Unit	Without FF	With FF	Reduction (%) [95% CI]
Without longitudinal feedback				
Absolute error in mean speed	mm/s	6.22	1.50	75.9 [74.2, 77.3]
Mean absolute travel-distance error	mm	48.5	15.1	68.8 [67.0, 70.8]
Velocity RMSE after the first 2 s	mm/s	6.56	2.63	60.0 [58.4, 61.4]
Full-segment velocity RMSE	mm/s	18.80	17.76	5.5 [4.9, 6.2]
Matched comparison with PI feedback				
Velocity RMSE after the first 2 s	mm/s	3.87	3.59	7.2 [0.5, 13.3]
Full-segment velocity RMSE	mm/s	16.19	16.18	0.1 [−0.9, 1.1]

## Data Availability

Dataset available on request from the authors.

## Supplementary Materials

The following supporting information can be downloaded at: https://www.mdpi.com/article/10.3390/s26217063/s1. Video S1: Physical experiments with feedforward compensation and integrated control. The first part displays two experiments with feedforward compensation without longitudinal feedback. The second part displays two experiments combining feedforward compensation, PI velocity feedback, and pure pursuit path tracking. The two experiments in each part are shown together.
